# Long-term cancer patient survival achieved by the end of the 20th century: most up-to-date estimates from the nationwide Finnish cancer registry

**DOI:** 10.1054/bjoc.2001.1905

**Published:** 2001-08

**Authors:** H Brenner, T Hakulinen

**Affiliations:** Department of Epidemiology, German Centre for Research on Ageing, Bergheimer Str. 20, Heidelberg, D-69115, Germany; Finnish Cancer Registry, Liisankatu 21 B, Helsinki, FIN-00170, Finland; Department of Public Health, University of Helsinki, Helsinki, FIN-00170, Finland

**Keywords:** cancer registry, prognosis, survival

## Abstract

A new method of survival analysis, denoted period analysis, has recently been developed, which has been shown to provide more up-to-date estimates of long-term survival rates than traditional methods of survival analysis. We applied period analysis to data from the nationwide Finnish cancer registry to provide up-to-date estimates of 5-, 10-, 15- and 20-year relative survival rates (RSR) achieved by the end of the 20th century. For most forms of cancer, period estimates of long-term survival are much higher than corresponding traditional survival estimates which suggests that for these cancers there has been ongoing major progress in survival rates in recent years which so far has remained undisclosed by traditional methods of survival analysis. For example, period analysis reveals that 10 year RSR have come close to (or even exceed) 80% for cancer of the corpus uteri and melanoma, 75% for breast cancer, 70% for bladder cancer, 65% for cancer of the cervix uteri, and 55% for cancer of the colon and prostate. Period analysis further reveals that 20 year RSR have now come close to (or even exceed) 75% for endometrial cancer and melanoma, 60% for breast cancer and cervical cancer, 55% for colon cancer and bladder cancer, and 40%–50% for cancer of the rectum, the ovaries, kidneys and nervous system. © 2001 Cancer Research Campaign http://www.bjcancer.com


British Journal of Cancer (2001) 85(3), 367–371
© 2001 Cancer Research Campaign
doi: 10.1054/ bjoc.2001.1905, available online at http://www.idealibrary.com on IDEAL®

http://www.bjcancer.com

### Long-term cancer patient survival achieved by the end of the 20th century: most up-to-date estimates from the nationwide Finnish cancer registry

**H Brenner[1] and T Hakulinen[2,3]**

[1]Department of Epidemiology, German Centre for Research on Ageing, Bergheimer Str. 20, D-69115 Heidelberg, Germany; [2]Finnish Cancer Registry, Liisankatu 21 B, FIN-00170 Helsinki, Finland; [3]Department of Public Health, University of Helsinki, FIN-00170 Helsinki, Finland

**Summary** A new method of survival analysis, denoted period analysis, has recently been developed, which has been shown to provide more up-to-date estimates of long-term survival rates than traditional methods of survival analysis. We applied period analysis to data from the nationwide Finnish cancer registry to provide up-to-date estimates of 5-, 10-, 15- and 20-year relative survival rates (RSR) achieved by the end of the 20th century. For most forms of cancer, period estimates of long-term survival are much higher than corresponding traditional survival estimates which suggests that for these cancers there has been ongoing major progress in survival rates in recent years which so far has remained undisclosed by traditional methods of survival analysis. For example, period analysis reveals that 10 year RSR have come close to (or even exceed) 80% for cancer of the corpus uteri and melanoma, 75% for breast cancer, 70% for bladder cancer, 65% for cancer of the cervix uteri, and 55% for cancer of the colon and prostate. Period analysis further reveals that 20 year RSR have now come close to (or even exceed) 75% for endometrial cancer and melanoma, 60% for breast cancer and cervical cancer, 55% for colon cancer and bladder cancer, and 40%–50% for cancer of the rectum, the ovaries, kidneys and nervous system. © 2001 Cancer Research Campaign http://www.bjcancer.com

**Keywords**: cancer registry; prognosis; survival

Long-term survival rates, such as 5-, 10- or 20-years survival rates are essential outcome measures of cancer, and they are now routinely reported by many cancer registries from different parts of the world. Unfortunately, traditional estimates of long-term survival, which pertain to cohorts of patients diagnosed many years ago, may be seriously outdated in case of recent improvement in survival. For example, in the 1999 report from the EUROCARE project, a collaborative effort of the population based European cancer registries to provide standardized data on cancer patient survival, 5-year survival rates were reported for patients diagnosed in 1985–1989 and followed with respect to survival until the end of 1994 (Berrino et al, 1999). Similarly, a recent analysis from the United States provided 5-, 10- and 15-year survival rates for patients diagnosed in 1974–1991 with a follow-up through 1992 (Wingo et al, 1998).

Recently, a new method of survival analysis, denoted period analysis, has been developed (Brenner and Gefeller, 1996), which has been shown to provide more up-to-date estimates of long-term survival rates (Brenner and Gefeller, 1997), but, with few exceptions (Brenner et al, 1998, 1999), the method has rarely been applied by cancer registries so far. We applied period analysis to data from the nationwide Finnish cancer registry to provide up-to-date estimates of long-term survival rates achieved by the end of the 20th century. In this paper, we present period estimates of 5-, 10-, 15- and 20-year relative survival rates pertaining to the 1995–1997 period (the most recent period for which complete data were available at the time of analysis) for the 16 most common forms of cancer, and we compare them with the corresponding survival estimates that might have been obtained from the same database by traditional methods of survival analysis.

*Received 15 December 2000*
*Revised 26 April 2001*
*Accepted 2 May 2001*

*Correspondence to:* H Brenner

### MATERIALS AND METHODS

### Data base

Our analysis is based on data from the nationwide Finnish Cancer Registry (population base: about 5.1 million people) which are among the highest quality data of any population-based cancer registry in the world. Virtually complete population-based cancer registration has been accomplished since 1953 (Teppo et al, 1994). Notification of cancer cases to the registry is mandatory by law, and it comes from many different sources, including hospitals, physicians working outside hospitals, dentists, and pathological and cytological laboratories. Copies are also obtained of all death certificates where cancer is mentioned.

Mortality follow-up is extremely efficient in Finland due to the existence of personal identification numbers (Dickman et al, 1999). Using these numbers as the key, the cancer registry files are matched annually with the annual list of deaths. Matching with the central population register (a register of all people currently alive and living in Finland) is performed as an additional check on the vital status of patients. By the time of this analysis, follow-up with respect to vital status had been completed until the end of 1997.

The current analysis includes patients diagnosed with one of the 16 most common forms of cancer (excluding non-melanoma skin cancer) in Finland between 1975 and 1997. Patients whose cancer was registered by death certificate only (about 2% of registered cases) or whose month of death was unknown (1.9%) were excluded from the analysis.

### Methods of analysis

Throughout this paper, we present relative rather than absolute survival rates (Ederer et al, 1961). Relative survival rates (RSR) are the preferred measures of survival reported by cancer registries, because they are unaffected by deaths from causes other than the primary cancer of interest. The RSR, which represents the survival rate in the hypothetical situation where the cancer in question is the only possible cause of death, is defined as the absolute survival rate among cancer patients divided by the expected survival rate of a comparable group from the general population. We estimated the expected survival rates from nationwide population life tables stratified by age, sex and calendar time according to the approach commonly known as the Ederer II method (with minor adaptations for the application of period analysis) (Ederer and Heise, 1959).

For each cancer site, the period estimates of 5-, 10-, 15- and 20-year RSR were obtained for the 1995–1997 period, the most recent 3-year period for which both registration of new cases and mortality follow up was complete at the time of analysis. Details of period analysis have been reported elsewhere (Brenner and Gefeller 1996, 1997). Briefly, the period estimates are obtained by left truncation of observations at the beginning of some recent period of interest (here: beginning of 1995) in addition to right censoring at its end (here: end of 1997). This ensures that period estimates, in contrast to traditional survival estimates, exclusively reflect survival experience during some recent time period (here: 1995–1997).

The most recent estimates of RSR obtained by traditional methods of survival analysis are reported for comparison. Traditional methods include ‘cohort analysis’ which focuses on cohorts of patients who have been under observation for the entire follow-up period of interest, and ‘complete analyses’ which additionally includes more recently diagnosed patients who have not completed the entire follow-up period of interest at the closing date of follow-up (here: end of 1997), but who are censored at that point of time. Survival figures reported by cancer registries so far have been derived either by pure forms of cohort or complete analysis, or by mixed forms of them (in that only patients who had been under observation for some minimum follow-up period were included).

Derivation of cohort, complete and period survival estimates presented in this paper and their differences are illustrated for 5-year survival rates in Figure 1. The most recent cohort estimate of 5-year survival pertains to patients diagnosed in 1990–1992 all of whom have completed 5-year follow-up by the end of 1997 (solid frame). In the derivation of the most recent period estimate, different parts of the survival function are obtained from the survival experience in 1995–1997 of patients diagnosed in different years (dashed frame). The complete estimates reflect the entire survival experience in 1990–1997 of patients diagnosed in 1990–1997, which includes the survival experience on which the cohort and the period estimates are based as overlapping sub-components.

| Calendar Years of diagnosis | Calendar Years of follow-up | | | | | | | |
|---|---|---|---|---|---|---|---|---|
| | 1990 | 1991 | 1992 | 1993 | 1994 | 1995 | 1996 | 1997 |
| 1990 | 1 | 1/2 | 2/3 | 3/4 | 4/5 | 5 | | |
| 1991 | | 1 | 1/2 | 2/3 | 3/4 | 4/5 | 5 | |
| 1992 | | | 1 | 1/2 | 2/3 | 3/4 | 4/5 | 5 |
| 1993 | | | | 1 | 1/2 | 2/3 | 3/4 | 4/5 |
| 1994 | | | | | 1 | 1/2 | 2/3 | 3/4 |
| 1995 | | | | | | 1 | 1/2 | 2/3 |
| 1996 | | | | | | | 1 | 1/2 |
| 1997 | | | | | | | | 1 |

**Figure 1** Survival experience included in the most up-to-date estimates of 5-year survival rates obtained by cohort analysis (solid frame), complete analysis (entire area), and period analysis (dashed frame). The figures 1–5 within the cells indicate the years of follow-up since diagnosis the particular cell defined by calendar years of diagnosis and follow-up is contributing to

For all methods, 95% confidence intervals of 5-year RSR, derived by Greenwood’s method (Greenwood, 1926) are provided along with the point estimates.

### RESULTS

Table 1 provides an overview on the types of cancer and the numbers of patients diagnosed in 1975–1997 who are included in this analysis. The most common forms of cancer were breast and lung cancer with an average of more than 2000 incident cases per year, followed by cancer of the prostate, stomach and colon. Analyses for the less common forms of cancer included in this paper, cancer of the esophagus and cervical cancer, are still based on an average number of about 200 cases per year. Table 1 also provides trends in 5-year relative survival rates (derived by cohort analysis) for patients diagnosed in various time intervals between 1975–1977 and 1990–1992. As expected, tremendous differences in prognosis between cancer sites were observed for patients diagnosed in each of the four time intervals, with the highest 5-year RSR for cancers of the breast and the corpus uteri and for melanoma, and very low 5-year RSR for cancers of the pancreas, oesophagus and lung. Unfortunately, the latter hardly changed over time. By contrast, there was major improvement in prognosis over time for most other forms of cancer. Improvements were most pronounced for cancers of the stomach, colon, breast, kidney, bladder, the nervous system and for melanoma and leukaemia. These trends underline the importance of approaches that provide the most up-to-date estimates of long term survival rates.

The most up-to-date estimates of 5-year RSR and their 95% confidence intervals derived by the different methods of survival analysis are presented in Table 2.

Whereas 5-year RSR estimates from cohort, complete and period analysis were rather similar for cancers of the oesophagus (7.5–7.7%), stomach (24.8–26.4%), pancreas (2.4–2.6%), lung (9.6–9.8%), nervous system (55.8–56.9%) and for melanoma (81.3–82.4%), there were major differences for other cancer sites (with the highest estimates obtained by period analysis and the lowest estimates obtained by cohort analysis). Differences were most salient for cancer of the uterine cervix (55.4–67.8%) and the prostate (63.5–72.6%), but major differences were also seen for cancer of the colon (52.7–57.6%), rectum (48.0–54.8%), ovaries (45.7–51.2%) and the bladder (68.7–75.3%) and for leukaemia (41.9–47.3%). Although 95% confidence intervals are somewhat

 *© 2001 Cancer Research Campaign*

**Table 1** Number of patients included in this analysis and trends in 5-year relative survival rates (RSR, in %) by type of cancer. Database: Finnish Cancer Registry, 1975–1997

| | | | 5-year RSR of patients diagnosed in | | | |
|---|---|---|---|---|---|---|
| **Cancer site** | **Sex** | ***n*** | **1975–77** | **1980–82** | **1985–87** | **1990–92** |
| Oesophagus | f+m | 4645 | 4.8 | 6.9 | 8.3 | 7.5 |
| Stomach | f+m | 24 901 | 12.9 | 17.9 | 19.7 | 24.8 |
| Colon | f+m | 20 483 | 38.6 | 47.3 | 47.4 | 52.7 |
| Rectum | f+m | 14 857 | 40.1 | 42.0 | 47.8 | 48.0 |
| Pancreas | f+m | 13 070 | 1.4 | 1.5 | 2.8 | 2.4 |
| Lung | f+m | 47 767 | 8.9 | 9.9 | 11.6 | 9.7 |
| Breast | f | 51 529 | 67.4 | 73.4 | 76.8 | 81.6 |
| Cervix uteri | f | 4033 | 60.4 | 57.5 | 56.2 | 55.5 |
| Corpus uteri | f | 11 617 | 74.9 | 75.9 | 75.1 | 80.3 |
| Ovary | f | 10 798 | 36.0 | 44.8 | 42.9 | 45.7 |
| Prostate | m | 31 355 | 52.4 | 55.2 | 60.5 | 63.5 |
| Kidney | f+m | 11 609 | 35.5 | 40.4 | 46.9 | 54.1 |
| Bladder | f+m | 13 916 | 52.1 | 62.3 | 64.2 | 68.7 |
| Melanoma | f+m | 9515 | 66.9 | 75.3 | 79.9 | 81.3 |
| Nervous system | f+m | 12 500 | 41.0 | 55.0 | 59.7 | 55.8 |
| Leukaemia | f+m | 8347 | 24.2 | 28.2 | 35.9 | 41.9 |

wider for estimates from cohort and period analyses than for estimates from complete analysis, the lower bounds of 95% confidence intervals of period estimates often exceed or are very close to the point estimates from complete analysis for those cancers with major differences in estimates from the three types of analysis.

The differences between the three types of estimates are generally more pronounced for 10-year RSR (see Table 3). For example, period estimates, different from the traditional estimates, disclose that 10-year RSR have now come close to (or even exceed) 80% for cancer of the corpus uteri and melanoma, 75% for breast cancer, 70% for bladder cancer, 65% for cancer of the cervix uteri, and 55% for cancer of the colon and prostate.

Still stronger differences between the different types of estimates are seen for 15- and 20-year RSR. In particular, 20-year period estimates of RSR are much higher than traditional 20-year estimates of RSR for all common cancer sites except those few cancers for which there has been virtually no improvement in prognosis over time and whose long-term prognosis has remained discouragingly poor, namely cancer of the pancreas and lung. For these cancers, the period estimates of 20-year RSR (1.9% and 3.7%, respectively) are very similar to the corresponding estimates obtained by traditional survival estimates. By contrast, period analysis suggests that 20-year RSR has now come close to (or even exceeds) 75% for endometrial cancer and melanoma, 60% for breast cancer and cervical cancer, 55% for colon cancer and bladder cancer, and 40–50% for cancer of the rectum, the ovaries, kidneys and nervous system. These estimates typically exceed the corresponding complete and cohort estimates by 5 to 15 per cent units and 10 to 20 per cent units, respectively.

A more comprehensive illustration of the major differences in the most up-to-date survival estimates obtained by the different methods of analysis is given by the 20-year relative survival curves, which are shown for breast cancer, the most common form of cancer among women, in Figure 2. For example, according to cohort and complete analysis, cumulative tumour associated mortality of 20% (corresponding to an RSR of 80%) is reached as early as about 2.5 and 4.5 years after diagnosis, respectively, compared to about 6.5 years according to period analysis. Cumulative tumour-associated mortality of 40% (corresponding to

**Table 2** Most up-to-date 5-year relative survival estimates (95% confidence intervals) for the most common forms of cancer according to cohort analysis, complete analysis and period analysis. Database: Finnish Cancer Registry, 1990–1997

| **Cancer site** | **Cohort analysis** | **Complete analysis** | **Period analysis** |
|---|---|---|---|
| Oesophagus | 7.5 (5.1–9.9) | 7.7 (5.8–9.6) | 7.7 (5.2–10.2) |
| Stomach | 24.8 (23.1–26.6) | 25.1 (23.8–26.5) | 26.4 (24.5–28.3) |
| Colon | 52.7 (50.5–54.9) | 54.7 (53.1–56.3) | 57.6 (55.4–59.7) |
| Rectum | 48.0 (45.4–50.6) | 51.2 (49.2–53.2) | 54.8 (52.1–57.4) |
| Pancreas | 2.4 (1.6–3.1) | 2.6 (2.0–3.3) | 2.6 (1.8–3.5) |
| Lung | 9.7 (8.9–10.5) | 9.8 (9.2–10.5) | 9.6 (8.7–10.4) |
| Breast | 81.6 (80.5–82.7) | 82.5 (81.7–83.4) | 83.4 (82.4–84.4) |
| Cervix uteri | 55.5 (50.0–61.0) | 60.8 (56.9–64.7) | 67.8 (62.7–72.8) |
| Corpus uteri | 80.3 (77.8–82.8) | 81.3 (79.6–83.1) | 82.4 (80.0–84.7) |
| Ovary | 45.7 (43.1–48.4) | 48.7 (46.7–50.6) | 51.2 (48.6–53.9) |
| Prostate | 63.5 (61.3–65.6) | 68.9 (67.2–70.5) | 72.6 (70.6–74.6) |
| Kidney | 54.1 (51.5–56.8) | 55.5 (53.6–57.5) | 58.3 (55.6–60.9) |
| Bladder | 68.7 (65.9–71.5) | 71.7 (69.6–73.7) | 75.3 (72.6–78.1) |
| Melanoma | 81.3 (78.6–84.0) | 81.5 (79.5–83.6) | 82.4 (79.8–85.1) |
| Nervous system | 55.8 (53.5–58.2) | 56.8 (55.1–58.4) | 56.9 (54.6–59.2) |
| Leukaemia | 41.9 (38.6–45.1) | 44.2 (41.8–46.6) | 47.3 (43.9–50.6) |

*© 2001 Cancer Research Campaign* 

**Table 3** Most up-to-date 10-, 15- and 20-year relative survival estimates for the most common forms of cancer according to cohort analysis, complete analysis and period analysis. Database: Finnish Cancer Registry, 1985–1997, 1980–1997 and 1975–1997, respectively

| | 10-year RSR | | | 15-year RSR | | | 20-year RSR | | |
|---|---|---|---|---|---|---|---|---|---|
| **Cancer site** | **Cohort** | **Complete** | **Period** | **Cohort** | **Complete** | **Period** | **Cohort** | **Complete** | **Period** |
| Oesophagus | 7.6 | 7.6 | 7.3 | 5.1 | 6.8 | 7.5 | 3.8 | 5.9 | 8.9 |
| Stomach | 17.7 | 21.3 | 24.3 | 15.0 | 18.5 | 23.3 | 8.9 | 14.2 | 21.0 |
| Colon | 43.1 | 48.9 | 55.6 | 42.0 | 46.5 | 54.2 | 32.4 | 43.5 | 53.5 |
| Rectum | 40.5 | 42.5 | 48.1 | 34.5 | 39.7 | 48.2 | 32.0 | 37.5 | 47.9 |
| Pancreas | 1.8 | 1.9 | 2.2 | 1.0 | 1.6 | 1.8 | 1.3 | 1.5 | 1.9 |
| Lung | 7.7 | 7.1 | 6.6 | 4.5 | 5.0 | 4.9 | 3.3 | 3.7 | 3.7 |
| Breast | 65.8 | 70.0 | 73.5 | 51.9 | 59.7 | 66.6 | 40.8 | 51.7 | 61.8 |
| Cervix uteri | 49.9 | 51.9 | 64.1 | 45.3 | 47.9 | 60.5 | 45.7 | 45.1 | 60.6 |
| Corpus uteri | 70.8 | 75.7 | 80.4 | 69.7 | 71.4 | 77.4 | 64.7 | 67.0 | 73.9 |
| Ovary | 38.6 | 42.2 | 46.0 | 36.7 | 37.9 | 42.0 | 27.7 | 35.4 | 41.9 |
| Prostate | 41.6 | 47.2 | 53.4 | 31.6 | 35.1 | 41.2 | 20.6 | 24.6 | 30.6 |
| Kidney | 38.9 | 45.0 | 50.3 | 25.5 | 35.9 | 46.1 | 22.3 | 32.3 | 42.5 |
| Bladder | 54.4 | 61.3 | 68.4 | 46.8 | 53.0 | 63.3 | 27.3 | 42.3 | 56.5 |
| Melanoma | 77.4 | 78.0 | 79.3 | 65.9 | 72.6 | 78.3 | 58.6 | 69.1 | 76.1 |
| Nervous system | 55.3 | 52.0 | 51.3 | 42.7 | 46.6 | 48.6 | 31.6 | 42.6 | 46.4 |
| Leukaemia | 25.3 | 29.7 | 35.3 | 14.5 | 21.1 | 28.5 | 10.3 | 16.0 | 23.7 |

an RSR of 60%) is reached about 7.5 years and 12.5 years after diagnosis according to cohort and complete analysis, respectively, whereas it remains below that level for more than 20 years according to period analysis.

### DISCUSSION

Monitoring of cancer survival rates is among the most important tasks of population-based cancer registries. To be useful for both clinical practice and public health purposes, estimates of survival rates should be as up-to-date as possible. It has previously been shown that changes in survival rates are more timely detected by period analysis, a recently introduced new method of survival analysis, than by traditional methods of survival analysis (Brenner and Gefeller, 1996, 1997). Our application of period analysis to the most recent survival data in the Finnish cancer registry confirms that the method of analysis matters indeed when one attempts to derive the most up-to-date long term survival estimates. For all but few forms of cancer, whose prognosis remained virtually unchanged over time, period analysis yielded substantially higher long-term survival estimates than the traditional methods of analysis.

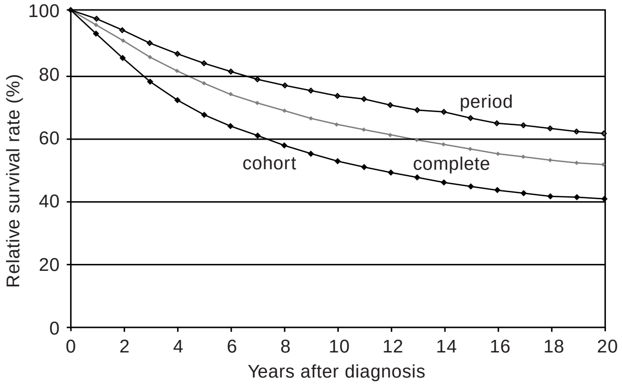

**Figure 2** Most up-to-date 20-year relative survival curves for breast cancer according to cohort analysis, complete analysis and period analysis. Database: Finnish Cancer Registry, 1975–1997

The differences in survival estimates obtained by the different types of analysis increase with increasing length of follow-up, and they are particularly strong for 10-, 15- and 20-year survival rates. The reason for that is that traditional long-term survival estimates, in contrast to period estimates, are strongly influenced by survival experience in the early years following diagnosis (when most cancer deaths occur) of patients diagnosed many years ago. They may thus be seriously outdated in case of recent major progress in survival, e.g. by advances in cancer therapy.

While the period approach has only recently been introduced in survival analysis, it is well established in other fields of science. For example, a widely used integrative (inverse) measure of total mortality over various ages is the life expectancy. The by far most commonly used measures to describe life expectancy achieved by the end of the 20th century are estimates from period life tables for some recent year (e.g. the year 1999), which reflect the mortality experience of people born at various parts of the century (who contribute mortality rates at various ages in 1999). One could also use a cohort approach to describe the life expectancy of the most recent cohort of people who has virtually died out by the end of the century (i.e., people born a lifespan ago), but these estimates would be dramatically lower as they would reflect mortality rates partly dating back a very long time ago.

Although period estimates of survival are more up-to-date than traditional estimates of survival, they still may lag behind the survival experience of newly diagnosed patients in the case of ongoing improvement in survival. To evaluate this issue further, we systematically compared the long-term survival rates actually observed for cohorts of Finnish cancer patients diagnosed in various time intervals between 1963 and 1992 with the most up-to-date estimates of long-term survival rates that might have been obtained from the Finnish Cancer Registry data at the time of diagnosis of these patients. This analysis gave a very consistent picture (data not shown): except for the few cancers, whose prognosis virtually remained unchanged over time, all types of survival analysis yielded ‘conservative’ estimates, i.e., estimates that were lower than those later observed for newly diagnosed patients, but in all cases, this discrepancy was much smaller for period analysis than for complete analysis and for cohort analysis.

 *© 2001 Cancer Research Campaign*

We therefore believe that the period estimates of long-term survival provided in this paper give a better picture of chances of long-term survival achieved by the end of the 20th century than previously available estimates that were based on traditional methods of survival analysis. According to our analysis, long-term survival rates are substantially higher than previously available figures for most forms of cancer indeed. For example, in the 1999 report of the EUROCARE project, cohort estimates of 5-year RSR were reported for patients diagnosed in 1985–1989 (Berrino et al, 1999). For the Finnish cancer patients, these cohort estimates were quite similar as, and in some cases (stomach cancer, ovarian cancer, kidney cancer and leukaemia) even substantially lower than the cohort estimates for the 1990–1992 cohorts presented in this paper. For all cancers except for those with virtually no improvement over time (cancer of the oesophagus, pancreas and lung and melanoma of the skin), however, the cohort estimates published in the 1999 EUROCARE report are substantially lower than the period estimates reported in this paper. Another recent comprehensive analysis of cancer patient survival in Finland reported also 10-year RSR (Dickman et al, 1999). In this analysis, 10-year RSR were derived for patients diagnosed in 1985–1994 and followed with respect to mortality until the end of 1995. This approach comes very close to a ‘pure form’ of complete analysis. Despite some variation due to the different years included in the analysis, results are quite close to the estimates from complete analysis reported in this paper, but they are substantially lower than estimates from period analysis for some forms of cancer with recent major improvement in prognosis (such as colorectal cancer, cancer of the cervix uteri and ovarian cancer).

For most forms of cancer, survival rates of cancer patients in Finland were somewhat higher than average survival rates from the European countries included in the recent EUROCARE report (Berrino et al, 1999). Nevertheless, there was a large variation between countries, and substantially higher rates were reported for some cancers from other countries. Likewise, the most recently reported survival estimates for white cancer patients in the United States (Greenlee et al, 2000) are often substantially higher than the survival rates reported from Finland so far (and even higher than the period estimates reported in this paper). These survival rates were also derived by traditional methods of survival analysis, and we therefore suspect that still substantially higher survival estimates would be obtained for these populations if period analysis was applied. We suggest to apply period analysis along with traditional techniques of survival analysis in comparative analyses of survival rates across registries in the future in order to obtain the most up-to-date possible picture of variation in survival rates across populations.

### REFERENCES

Berrino F, Cappocaccia R, Estève J, Gatta G, Hakulinen T, Micheli A, Sant M and Verdecchia A (eds) (1999) *Survival of cancer patients in Europe: The EUROCARE-2 Study*. IARC Scientific Publication 151. IARC: Lyon

Brenner H and Gefeller O (1996) An alternative approach to monitoring cancer patient survival. *Cancer* **78**: 2004–2010

Brenner H and Gefeller O (1997) Deriving more up-to-date estimates of long term patient survival. *J Clin Epidemiol* **50**: 211–216

Brenner H, Stegmaier C and Ziegler H (1998) Recent improvement in survival of breast cancer patients in Saarland/Germany. *Brit J Cancer* **78**: 694–697

Brenner H, Stegmaier C and Ziegler H (1999) Trends in survival of patients with ovarian cancer in Saarland, Germany, 1976–1995. *J Cancer Res Clin Oncol* **125**: 109–113

Dickman PW, Hakulinen T, Luostarinen T, Pukkala E, Sankila R, Söderman B and Teppo L (1999) Survival of cancer patients in Finland 1955–1994. *Acta Oncol 38 Suppl* **12**: 1–103

Ederer F and Heise H (1959) Instructions to IBM 650 programmers in processing survival computations. Methodological note No. 10, End Results Evaluation Section. National Cancer Institute: Bethesda (MD)

Ederer F, Axtell LM and Cutler SJ (1961) The relative survival rate: a statistical methodology. *Natl Cancer Inst Monogr* **6**: 101–121

Greenlee RT, Murray T, Bolden S and Wingo PA (2000) Cancer statistics, 2000. *CA Cancer J Clin* **50**: 7–33

Greenwood M (1926) A report on the natural duration of cancer. Ministry of Health, His Majesty’s Stationery Office: London

Teppo L, Pukkala E and Lehtonen M (1994) Data quality and quality control of a population based cancer registry. Experience in Finland. *Acta Oncol* **33**: 365–369

Wingo PA, Gloeckler Ries LA, Parker SL and Heath CW (1998) Long-term cancer patient survival in the United States. *Cancer Epidemiol Biomark Prev* **5**: 247–251

*© 2001 Cancer Research Campaign*
